# Synergetic Effect of 4-Phenylbutyric Acid in Combination with Cyclosporine A on Cardiovascular Function in Sepsis Rats via Inhibition of Endoplasmic Reticulum Stress and Mitochondrial Permeability Transition Pore Opening

**DOI:** 10.3389/fphar.2021.770558

**Published:** 2021-11-30

**Authors:** Lei Kuang, Yu Zhu, Yue Wu, Xiaoyong Peng, Kunlun Tian, Liangming Liu, Tao Li

**Affiliations:** Department of Shock and Transfusion, State Key Laboratory of Trauma, Burns and Combined Injury, Daping Hospital, Third Military Medical University (Army Medical University), Chongqing, China

**Keywords:** sepsis, cardiovascular function, synergetic effects, endoplasmic reticulum stress, mitochondrial permeability transition pore opening

## Abstract

**Background:** Sepsis/septic shock is a common complication in the intensive care unit, and the opening of the mitochondrial permeability transition pore (mPTP), as well as the endoplasmic reticulum stress (ERS), play important roles in this situation. Whether the combination of anti-ERS and anti-mPTP by 4-phenylbutyric acid (PBA) and Cyclosporine A (CsA) could benefit sepsis is unclear.

**Methods:** The cecal ligation and puncture-induced septic shock models were replicated in rats, and lipopolysaccharide (LPS)-challenged primary vascular smooth muscle cells and H9C2 cardiomyocytes *in vitro* models were also used. The therapeutic effects of CsA, PBA, and combined administration on oxygen delivery, cardiac and vascular function, vital organ injury, and the underlying mechanisms were observed.

**Results:** Septic shock significantly induced cardiovascular dysfunction, hypoperfusion, and organ injury and resulted in high mortality in rats. Conventional treatment including fluid resuscitation, vasoactive agents, and antibiotics slightly restored tissue perfusion and organ function in septic rats. Supplementation of CsA or PBA improved the tissue perfusion, organ function, and survival of septic shock rats. The combined application of PBA and CsA could significantly enhance the beneficial effects, compared with using PBA or CsA alone. Further study showed that PBA enhanced CsA-induced cardiovascular protection, which contributed to better therapeutic effects.

**Conclusion:** Anti-ERS and anti-mPTP-opening by the combination of PBA and CsA was beneficial to septic shock. PBA enforced the CsA-associated cardiovascular protection and contributed to the synergetic effect.

## Introduction

Sepsis/septic shock is a common and severe complication in the intensive care unit. Despite substantial advances in the development of medicine and therapeutics, the mortality of severe sepsis remains high (Cecconi et al., 2018). Studies have demonstrated that tissue hypoperfusion and/or organ dysfunction play critical roles in severe sepsis- or septic shock-associated morbidity and mortality. Impairment in tissue hypoperfusion or oxygen availability induced by hemodynamic disturbances in the macro- and micro-circulatory are considered to be the main reasons for organ dysfunction following severe sepsis and septic shock (Zanotti-Cavazzoni and Hollenberg, 2009). The measures of protecting cardiovascular dysfunction may be the ideal treatment for sepsis.

Mitochondria are the center of energy metabolism and play important roles in the regulation of cell functions. Studies suggested that the degree of mitochondrial dysfunction in vital organs is associated with organ injury and mortality following sepsis (Pool et al., 2018; Supinski et al., 2020). Mitochondrial permeability transition pore (mPTP) is a nonspecific pore that plays important roles in the regulation of mitochondrial structure and function. Upon mPTP opening, free passage of protons across the inner membrane leads to dissipation of the membrane potential and pH gradient which comprise oxidative phosphorylation. mPTP opening results in disturbances in energy metabolism and cell damage (van Gurp et al., 2003; Leung and Halestrap, 2008). Several studies have demonstrated that mPTP opening participates in cardiovascular damage after ischemia-reperfusion, and anti-mPTP opening with Cyclosporine A (CsA) has a beneficial effect on ischemia-reperfusion injury in the heart, but its effect is limited (Morciano et al., 2017). We want to know whether other measures could enhance the beneficial effect of CsA.

Some studies have suggested that endoplasmic reticulum stress (ERS) contributed to mPTP opening. ERS stimulator, streptovirudin, leads to dephosphorylation of AKT and its target glycogen synthase kinase-3β which results in cardiac dysfunction, and these effects are antagonized by CsA (Zhang et al., 2013). Other studies demonstrated that ERS has important roles in the development of heart failure, hypertension, and atherosclerosis (Hong et al., 2017) and sepsis-induced cardiovascular dysfunction (Khan et al., 2015). Hence, we hypothesize that anti-ERS combined with CsA is beneficial to septic shock via enhancing cardiovascular protection.

To test this hypothesis, cecal ligation and puncture (CLP)-induced septic shock rats, as well as lipopolysaccharide (LPS)-treated vascular smooth muscle cells (VSMCs) and H9C2 cardiomyocyte cell line, were employed. The synergetic effects of CsA in combination with PBA on septic shock and the underlying mechanism were observed.

## Materials and Methods

### Ethical Approval of the Study

The study was approved by the Laboratory Animal Welfare and Ethics Committee of Third Military Medical University (Army Medical University) according to the guidelines of the ethical use of animals. The protocol conformed to the guidelines of the ethical use of animals. The investigation conformed to the Guide for the Care and Use of Laboratory Animals published by the US National Institutes of Health (NIH Publications, eighth edition, 2011), and all rats were guaranteed to suffer the least amount.

### Animals and Materials

A total number of 296 male and female Sprague–Dawley rats (220–240 g) were obtained from the Animal Center of Army Medical Center. They were maintained in a room at constant humidity (60 ± 5%), temperature (24 ± 1°C), and light cycle (6 am–6 pm) and fed standard pellet diets ad libitum. 4-Phenylbutyric acid (PBA, #P21005), Cyclosporin A (CsA, #SML1018), betulinc acid (Betu, #B8936), and Calcein-AM (#17783) were purchased from Sigma-Aldrich; MitoTracker DeepRed FM (#M22426) was purchased from Thermo Fisher Scientific. Anti-ANT1 antibody (#ab110322), anti-GRP78 antibody (#ab21685), and GRP94 antibody (#ab238126) were purchased from Abcam. Anti-VDAC1 antibody (#A19707) and anti-CypD (Cyclophilin 40) antibody (#A5097) were purchased from Abclonal. Anti-CHOP antibody (#2895) was purchased from Cell Signaling Technology. Anti-β-actin antibody (#MA1-744) and anti-GAPDH (#A21994) antibodies were purchased from Thermo Fisher Scientific.

### Septic Shock Rat Model Establishment

Rats were anaesthetized with sodium pentobarbital (30 mg/kg, i.p.) before operation. After sterilization, the cecum was exposed and ligated by 7.5 mm to its end. The ligated cecum was punctured (≈1.5 mm) with a triangular needle (sham-operated rats only received cecum ligation and no puncture). Feces were allowed to flow into the abdominal cavity. After the closure of the abdomen, rats were returned to their cages and allowed food and water ad libitum. Twelve hours after surgery, rats were re-anaesthetized and the femoral artery was catheterized (PE9050, inner diameter = 0.5 mm, SDR scientific), and mean arterial pressure (MAP) was measured. If MAP was <70 mmHg or if it decreased by >30% (compared with the sham-operated level), septic shock model was considered replicated. The success rate of septic shock was 89.2%.

### Fluid Therapy

Septic shock rats were randomly assigned into 4 groups receiving conventional therapy (CT), CsA (anti-mPTP opening), PBA (anti-ERS), and CsA + PBA. Conventional therapy consists of infusion of lactated Ringer’s solution (LR) through the right femoral vein, dopamine supplementation whenever necessary to maintain MAP >70 mmHg, and antibiotic (cefuroxime sodium, 50 mg/kg i.m.). Fluid was infused within 3 h and the total fluid volume was 30 ml/kg, and the maximum rate of dopamine infusion was ≤10 μg/kg/min. CsA (5 mg/kg) and PBA (5 mg/kg) were dissolved in LR and administered during fluid therapy. The following parameters were collected 6 h after infusion.

### Blood Flow and Organ Function

The tissue blood flow of the intestine and kidney were measured by a laser Periflux System 5,000 Doppler system (Perimed, Sweden) as described in our lab previously (Li et al., 2013). The abdomen cavity was opened and the probes were placed on the surface of vital organs. Biochemical variables including blood levels of d-lactate were measured with commercially available kits (Nanjing Jiancheng Bioengineering Institute, #H263), blood endotoxin, blood urea nitrogen (BUN), and serum creatinine (Scr) were measured by a DX800 Biochemical Analyzer (Beckman Coulter, United States). The volume of blood samples for measurement of biochemical analysis was 1.0–1.5 ml.

### Hemodynamic Parameters, Oxygen Delivery, and Utilization

The cardiac output (CO) was measured with thermodilution techniques as described previously (Liu et al., 2016). A thermodilution probe was inserted into the aorta ascendens of the rat through the right carotid artery, and 0.3 ml ice-bathed saline was injected through the right external jugular vein catheter. The CO was determined using a CO analyzer (PowerLab, AD Instruments, Australia). Arterial and venous blood gases were measured using a pHOx^®^ plus L Blood Gas Analyzer (Nova Biomedical, United States) with blood from the femoral artery and femoral vein. The volume of blood samples for measurement of blood gases was 0.3 ml and to avoid additional blood loss in rats, equal volumes of blood from donor rats were infused back after each sample was taken. Oxygen delivery (DO_2_) and oxygen consumption (VO_2_) were calculated using the following equations:

$${\text{DO}}_{2}=\text{CI×}13\text{.}4\text{×}\left[\text{Hb}\right]{\text{×SaO}}_{2}$$

$${\text{VO}}_{2}=\text{CI×}13\text{.}4\text{×}\left[\text{Hb}\right]\text{×}\left({\text{SaO}}_{2}{\;-\text{SvO}}_{2}\right)$$

CI in the equations is the cardiac index, [Hb] is the hemoglobin concentration, SaO_2_ is the oxygen saturation of the artery, and SvO_2_ is the oxygen saturation of the vein (Li et al., 2011a).

### Contractile Tension of Cardiac Muscle

The contractile tension of cardiac muscle was measured with the Powerlab system (AD Instruments, Australia) (Li et al., 2010a). Briefly, a small bundle of cardiac papillary muscle (CPM, diameter <1mm; length ≈5 mm) was dissected from the right ventricle. Then, it was mounted and suspended between the fixation hooks, and then immersed in an isolated organ chamber (Scientific Instruments, Spain) containing 37°C Krebs-Henseleit (K-H) solution (mmol/L): NaCl 120, KCl 4.7, NaH_2_PO_4_ 1.2, MgSO_4_ 1.2, CaCl_2_ 2.5, NaHCO_3_ 20, glucose 10 (pH 7.4) bubbled continuously with 95% O_2_/5% CO_2_. The CPM was given 1.0 g of preload for 60 min, and then received an electric stimulus (0.1 mV; frequency, 0.2 Hz; duration, 10 ms). The CPM was perfused with isoprenaline (ISO) of a series of concentrations (1×10^−10^–1×10^−4^ mol/L) and the contractile tension of CPM at each concentration of ISO was determined.

### Vascular Reactivity of Superior Mesenteric Artery

Vascular contractile and dilative function was expressed as the responsiveness of arteries to the norepinephrine (NE) or acetylcholine (Ach), as described previously (Zhu et al., 2013). The response of arteries to NE was measured by the PowerLab system via a force transducer (AD Instruments, Australia). SMA rings (diameter, 2–3 mm) were mounted on wire and suspended between a force transducer and a post attached to a micrometer, then immersed into an isolated organ chamber containing K-H solution. After equilibration for 2 h, the contractile responses of arterial rings to NE (1×10^−10^–1×10^−4^ mol/L) were measured. The dilative responses of arterial rings to Ach (1×10^−10^–1×10^−4^ mol/L) were measured subsequently.

### Cell Culture and Transfection

VSMCs were obtained from the mesenteric arteries of SD rats by enzymatic digestion as described previously in our research team (Li et al., 2010b; Li et al., 2011b). Briefly, isolate the whole mesenteric bed and place it in cold complete DMEM high glucose medium (Gibco, 12430047), clean the mesenteric bed by carefully removing the fat, incubate the mesenteric bed in a centrifuge tube containing the pre-digestion mix (DMEM, with collagenase type I from Sangon, #A004194) for 30 min, homogenize the vascular bed by syringing with 20G needle four times, filter cells and tissue debris through a 100 μm nylon filter, and collect the cell solution. Centrifuge the cell solution and resuspend the cell pellet in 5 ml of DMEM with 10% FBS (Gibco, 10099141) and culture the cells in a 37°C/5% CO_2_ incubator. Keep changing the medium every 2 days until cells reach confluence and are ready for either further culture. Rat cardiomyocyte cell line (H9C2) was obtained from ATCC and grown in DMEM high glucose and supplemented with 10% FBS. Before each experiment, cells were serum-starved for 12 h.

CHOP siRNA (GGA AAC GAA GAG GAA GAA UTT; AUU CUU CCU CUU CGU UUC CTT) and Lipofectamine 3,000 (Invitrogen, L3000015) were used for decreasing CHOP expression. Cells were seeded at 2 × 10^5^ cells/well on 6-well plate or 35 mm confocal dish ∼24 h before transfection. Transfections were performed in OPTI-MEM media (Gibco, 31985062) with 3 uL and 5 μL Lipofectamine 3,000 per well/dish for primary VSMCs and H9C2 cells, respectively, and 3 μg of siRNA were used per well/dish. The expression of the target protein was detected by immunoblotting or confocal microscope 48–72 h post-transfection.

### Mitochondrial Permeability Transition Pore Opening Observation

The extent of opening of the mPTP was determined by the calcein–Co^2+^ method using laser scanning confocal microscopy, as described previously (Duan et al., 2020). Briefly, VSMCs or H9C2 cardiomyocytes were seeded in 35 mm petri dishes and incubated with 2 µM calcein-AM and 100 nM MitoTracker DeepRed for 30 min and then exposed to 2 mM CoCl_2_ for 15 min at room temperature. Cells were then observed with a Leica TCS SP5 confocal system (Leica Microsystems, Germany), excited at 488 nm (calcein-AM) and 633 nm (MitoTracker Deep Red). When the mPTP opened, calcein-AM flowed out from mitochondria and fluorescence decreased. The mPTP opening was indicated by the decreased fluorescence intensity of calcein.

### Immunoblotting

Myocardium, superior mesenteric artery, VSMCs, and H9C2 cardiomyocytes were harvested and lysed, and protein extracts were separated by SDS-PAGE and transferred to nitrocellulose filter membrane, then immunoblotted with corresponding antibodies at a dilution of 1:1,000 (VDAC, CypD, ANT, CHOP, GRP78, GRP94, β-actin) and IRDye secondary antibodies (Li-Cor, United States) and analyzed with Odyssey CLx Imaging System (Li-cor, United States).

### Statistical Analyses

Parametric data were presented as the mean ± standard deviation of n observations. The statistical differences among groups were analyzed by ANOVA analysis, followed by the post-hoc Tukey test for multiple comparisons between groups. Survival was analyzed by Kaplan-Meier survival analysis and the log-rank test. *p* < 0.05 was considered significant.

## Results

### Synergetic Effects of PBA and CsA on Improving Animal Survival, Oxygen Delivery, and Utilization, Blood Gas Following Sepsis in Rats

**FIGURE 1:**
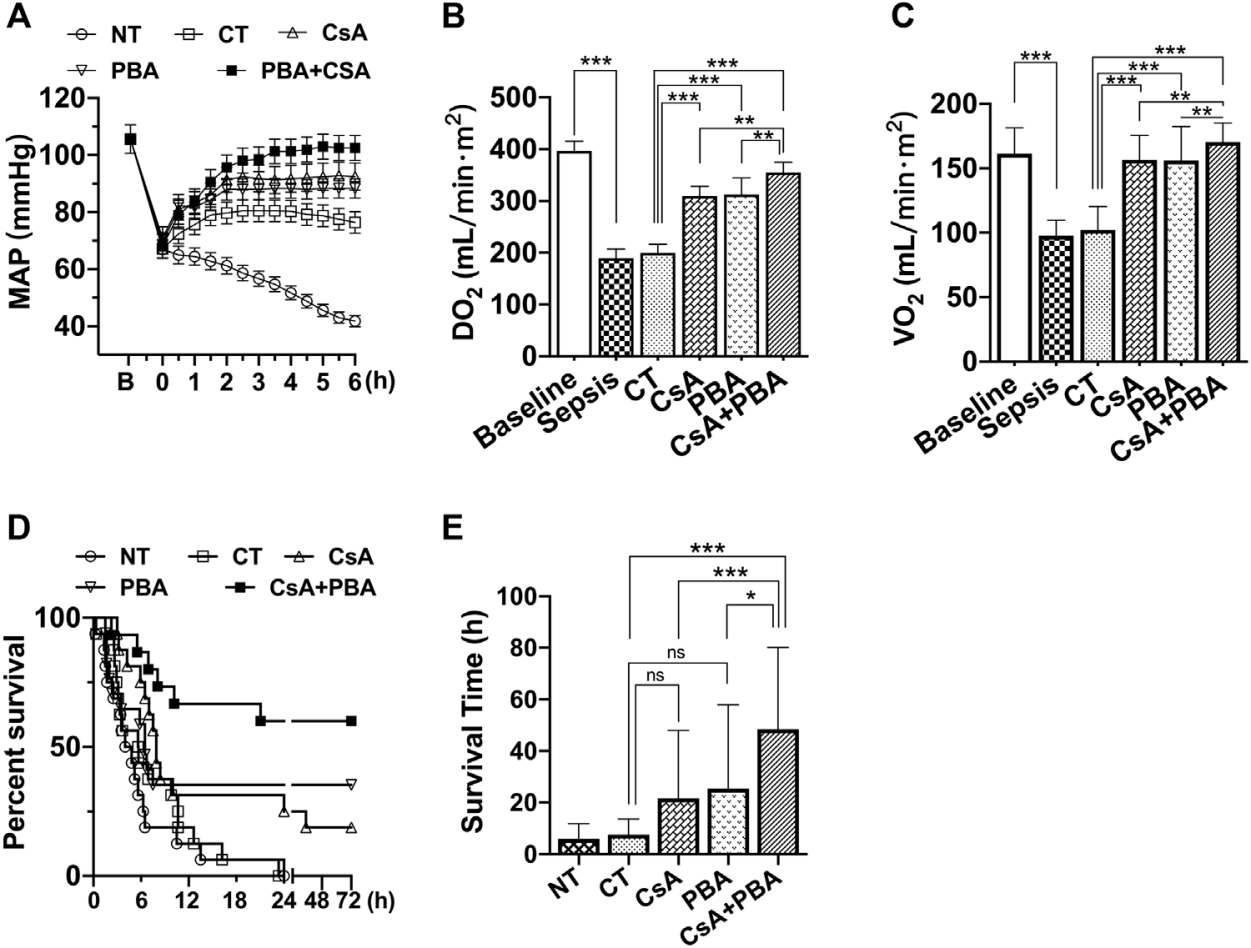
Synergetic effect of PBA and CsA on improving survival and oxygen delivery in CLP-induced septic shock rats. **(A)**, mean arterial pressure (MAP) (*n* = 8/group); **(B)**, oxygen delivery (DO_2_) (*n* = 8/group); **(C)**, oxygen utilization (VO_2_) (*n* = 8/group); **(D)**, survival rate and survival time (*n* = 16/group). CsA, cyclosporine A; PBA, 4-phenylbutyrate; CT, conventional treatment; NT, no treatment. *p < 0.05, **p < 0.01, ***p < 0.001.

The results showed that after septic shock, the MAP of rats was significantly decreased. Conventional therapy (CT) could increase and maintain MAP to about 80 mmHg in rats during fluid infusion, and then MAP tended to decrease after infusion. Supplementation of CsA or PBA alone was able to maintain MAP at about 88 ± 4.2 mmHg and 92 ± 4.8 mmHg, and combined administration of PBA and CsA significantly improved the MAP (102 ± 4.4 mmHg), compared with using PBA or CsA alone (Figure 1A).

CT moderately restored PaO_2_, SaO_2_, SvO_2_; however, the oxygen delivery (DO_2_) and utilization (VO_2_) were not increased. Supplementation of CsA or PBA alone significantly increased PaO_2_, SaO_2_, SvO_2_, DO_2_, and VO_2_, PBA, and CsA demonstrated similar effects. Combined administration of PBA and CsA significantly improved DO_2_, VO_2_, compared with PBA or CsA alone (Figures 1B,C and Table 1).

**TABLE 1:** Synergetic effects of PBA + CsA on blood gas following sepsis.

Group	Baseline	Sepsis	6 h after Treatment
PaO_2_ (mmHg)			
NT	99.2 ± 8.6	93.6 ± 8.8	88.9 ± 10.5
CT			94.7 ± 10.2
CsA			96.7 ± 10.8
PBA			97.3 ± 7.9
CsA + PBA			99.4 ± 5.3
SaO_2_ (%)			
NT	98.8 ± 0.9	91.3 ± 1.8	84.2 ± 1.4
CT			92.8 ± 2.5
CsA			96.4 ± 2.2*
PBA			95.4 ± 1.5^@^
CsA + PBA			99.3 ± 1**
SvO_2_ (%)			
NT	58.7 ± 3.9	44.1 ± 4	42.4 ± 3.7
CT			45.4 ± 6.1
CsA			47.5 ± 5.1
PBA			47.8 ± 5.4
CsA + PBA			51.6 ± 4.4*

CLP, cecal ligation and puncture; NT, no treatment; CT, conventional therapy; CsA, cyclosporine A; PBA, 4-phenylbutyrate acid. PaO_2_, partial pressure of oxygen in artery; SaO_2_ and SvO2, arterial and venous oxygen saturation. *p < 0.05, **p < 0.01 vs CT; ^@^ *p* < 0.05, vs PBA + CsA.

The animal survival time and the survival rate were significantly decreased following sepsis. Conventional therapy slightly improved animal survival (although not significant). Besides, in the PBA + CsA group, the 72 h survival rate of rats was 9/16, while it was only 3/16 in the CsA group and 4/16 in the PBA group. The average survival time was also significantly increased with the combined administration of CsA and PBA (Figures 1D,E).

### Synergetic Effects of PBA and CsA on Increasing Tissue Perfusion and Organ Function Following Sepsis in Rats

**FIGURE 2:**
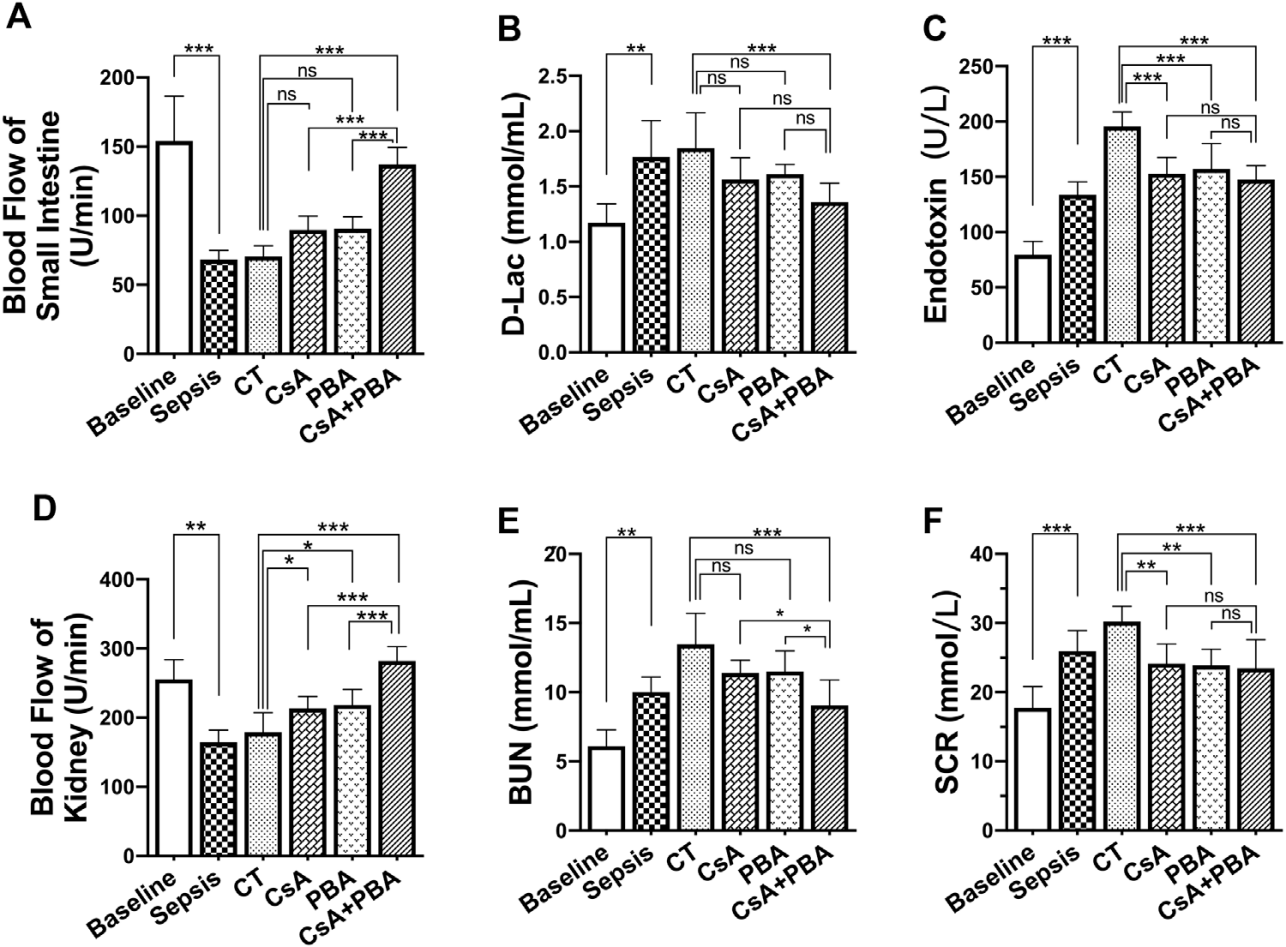
Synergetic effect of PBA and CsA on improving tissue perfusion and organ function in CLP-induced septic shock rats. (*n* = 8/group) **(A)**, the blood flow of intestine; **(B-C)**, intestinal barrier injury indices (d-lactate and endotoxin); **(D)**, the blood flow of kidney; **(E-F)**, renal function (blood urea nitrogen and serum creatinine). CsA, cyclosporine A; PBA, 4-phenylbutyrate acid; CT, conventional treatment. *p < 0.05, **p < 0.01, ***p < 0.001.

To further clarify the role of CsA, PBA, and the combined administration in septic shock, we observed the changes in tissue blood flow and organ function before and after infusion in septic shock rats. The results showed that the tissue blood flow of the kidney and small intestine were significantly decreased after septic shock in rats compared with sham-operated rats (Figures 2A,D). Conventional therapy failed to improve the intestinal or renal perfusion following septic shock. CsA or PBA slightly improved the blood flow of the kidney and tended to increase the blood flow of the small intestine. CsA + PBA significantly increased blood perfusion of intestine and kidney, compared with the CsA group, and the average blood flow of kidney and intestine were increased by 32.1 and 52.8%, respectively. The organ functions were significantly impaired after septic shock, and it was shown that the level of blood urine nitrogen, serum creatinine, d-lactate, and endotoxin were significantly increased (Figures 2B,C,E,F). Conventional therapy demonstrated limited effects in restoring organ injury. CsA or PBA alleviated the damage of organs. The combination of PBA with CsA significantly reduced the damage of organ function as compared with CsA or PBA alone.

### Phenylbutyric Acid and Cyclosporine A Synergetically Induced the Improvement of Cardiovascular Function Following Septic Shock

Cardiovascular dysfunction directly impairs tissue perfusion. Previous studies demonstrated that cardiovascular function was decreased after sepsis or septic shock, the damage degree of cardiovascular function was positively correlated with mortality of septic shock. In this section, the effects of the combination of PBA and CsA on cardiovascular function including the cardiac and vascular contractile reactivity, cardiac output, and cardiac index were investigated.

**FIGURE 3:**
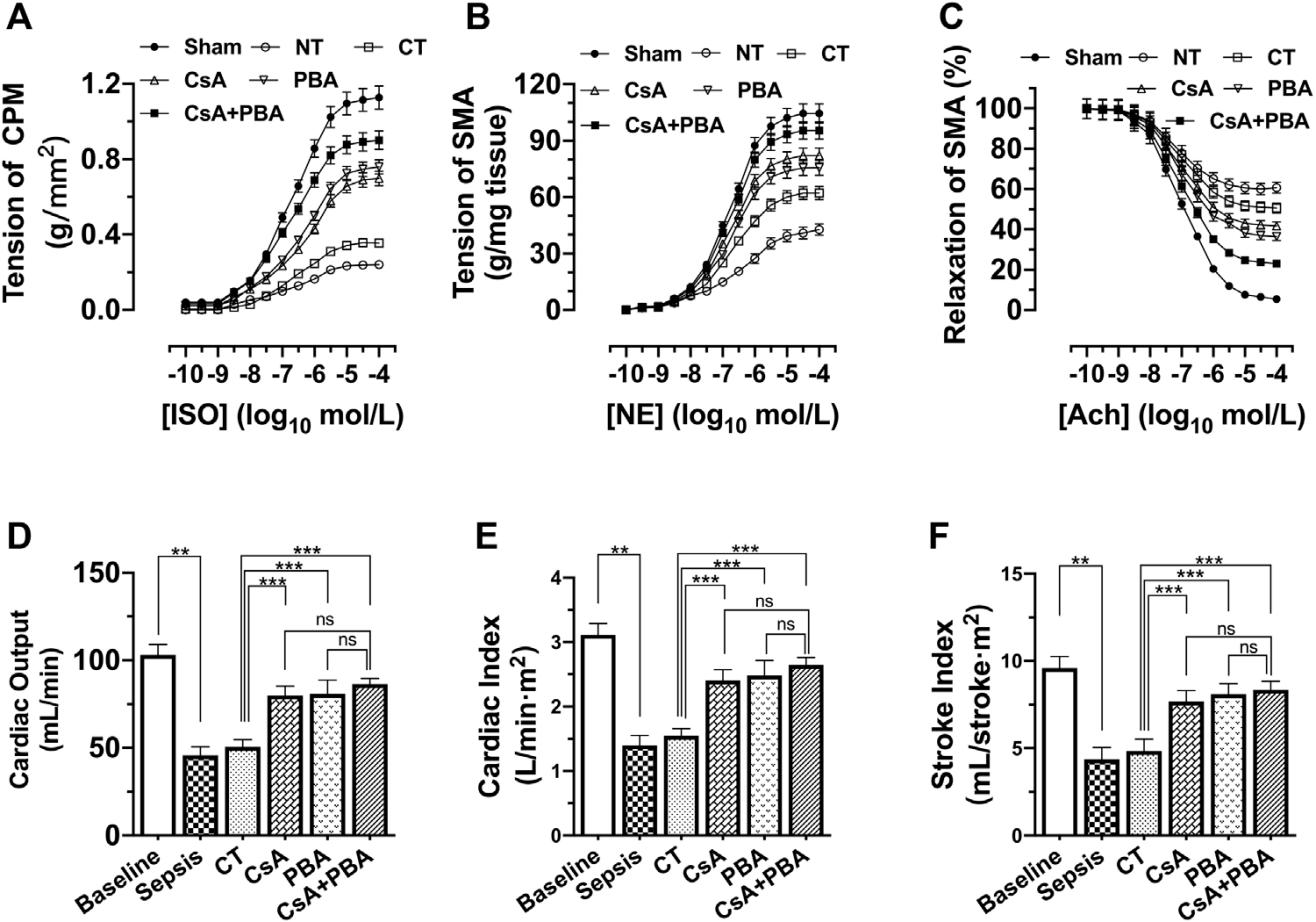
Synergetic effect of PBA and CsA on improving cardiovascular function after septic shock in rats. (*n* = 8/group). **(A)**, the contractile response of cardiac papillary muscle (CPM) to ISO; **(B)**, contractile response superior mesenteric artery to NE; **(C)**, relaxation response of superior mesenteric artery to Ach; **(D)**, cardiac output (CO); **(E)**, cardiac index (CI); **(F)**, stroke index (SI). CsA, cyclosporine A; PBA, 4-phenylbutyrate acid; CT, conventional treatment; ISO, isoprenaline; NE, norepinephrine; Ach, acetylcholine. *p < 0.05, **p < 0.01, ***p < 0.001; *ns*, not significant.

The results showed that the contractile forces of CPM and SMA to catecholamines (ISO and NE), and the cardiac output, cardiac index, and stroke index were significantly decreased in septic shock rats compared with sham-operated rats. Conventional therapy, CsA, or PBA alone improved the contractility of CPM and SMA. And the combined administration of PBA and CsA significantly improved cardiovascular function. PBA + CsA increased the maximal contraction of CPM and SMA to catecholamines by 29.18% and 16.28% and improved relaxation of SMA to acetylcholine by 37.29% (Figures 3A–C), compared with CsA alone. Besides, the cardiac output, cardiac index, and stroke index were significantly increased by CsA, PBA, and combined application, compared with LR. And the combination of CsA and PBA tended to demonstrate better therapeutic effects (Figures 3D–F).

### Effects of Cyclosporine A and Phenylbutyric Acid on Mitochondrial Permeability Transition Pore and Endoplasmic Reticulum Stress in H9C2 cardiomyocytes and Vascular Smooth Muscle Cells after Septic Shock

**FIGURE 4:**
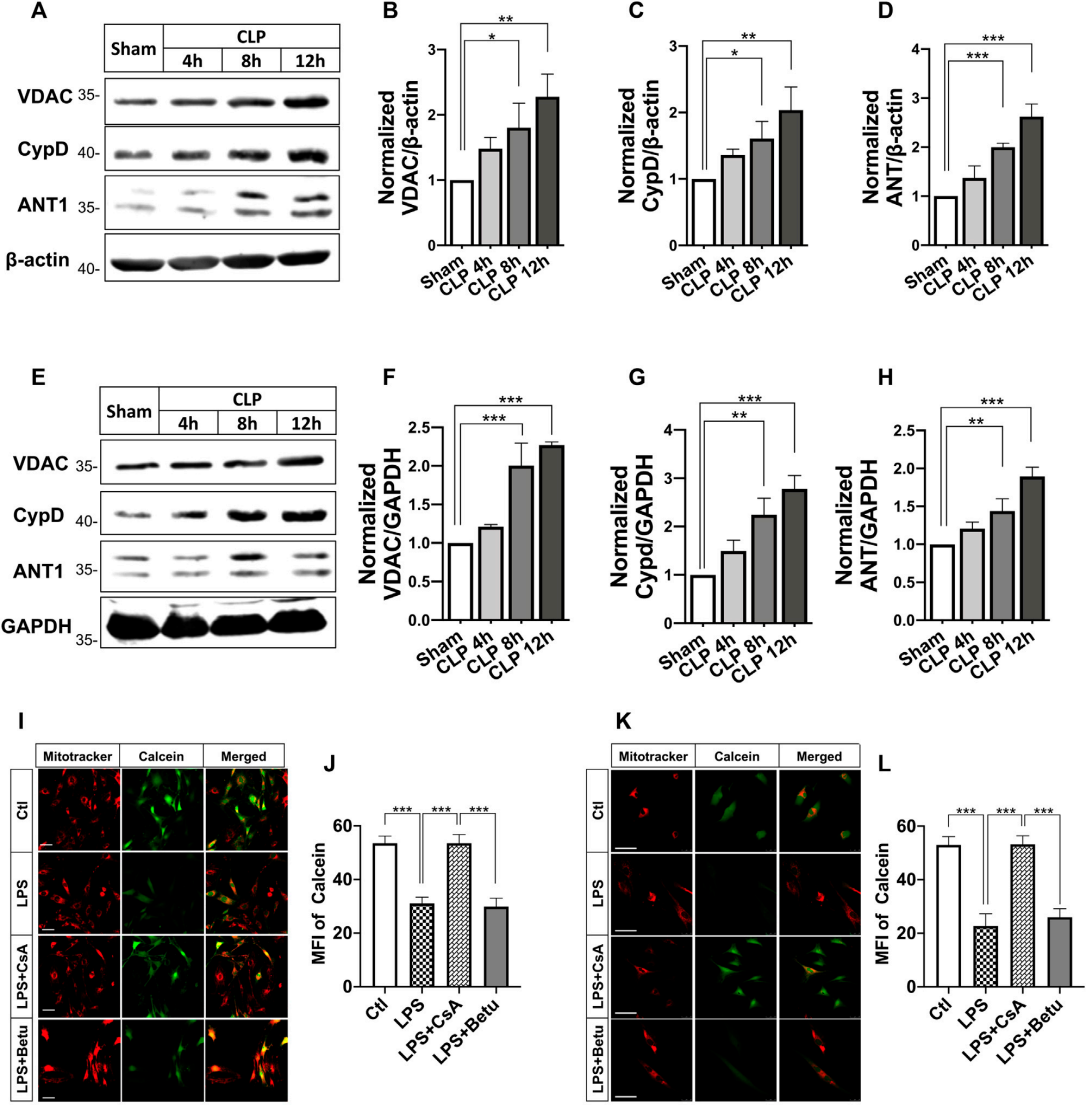
Effects of CsA on mPTP opening after septic shock in rats. **(A-C)**, expression of voltage-dependent anion channel (VDACs) and cyclophilin-D (CypD) and adenine nucleotide translocase (ANT) in the superior mesenteric artery (SMA) from septic-shock rats (*n* = 3); **(E-H)**, Expression of voltage-dependent anion channel (VDACs) and cyclophilin-D (CypD) and adenine nucleotide translocase (ANT) in the cardiac papillary muscle (CPM) from septic-shock rats (*n* = 3); **(I-L)**, mPTP opening in VSMCs and H9C2 cardiomyocytes (*n* = 20/group from 3 independent experiment). Scale bar = 50 μm. CLP, cecal ligation and puncture; CsA, cyclosporine A; Betu, betulinc acid; LPS, lipopolysaccharide; MFI, mean fluorescence intensity. *p < 0.05, **p < 0.01, ***p < 0.001; *ns*, not significant.

In this part, we observed the changes of the protein level of adenine nucleotide translocase1 (ANT1), cyclophilin 40 (CypD), as well as voltage-dependent anion channel 1 (VDAC1), which were regarded as mPTP components and regulators. The results indicated that the expressions of ANT1, VDAC, and CypD were significantly increased in SMA and heart tissue after septic shock. (Figures 4A–H). LPS incubation (500 ng/ml, 12 h) caused significant mPTP opening in VSMCs and H9C2 cardiomyocytes, while betulinc acid (Betu), an mPTP opener, demonstrated comparable effects at 1 μg/ml CsA (1 μmol/L) and significantly reduced mPTP opening (Figure 4I).

**FIGURE 5:**
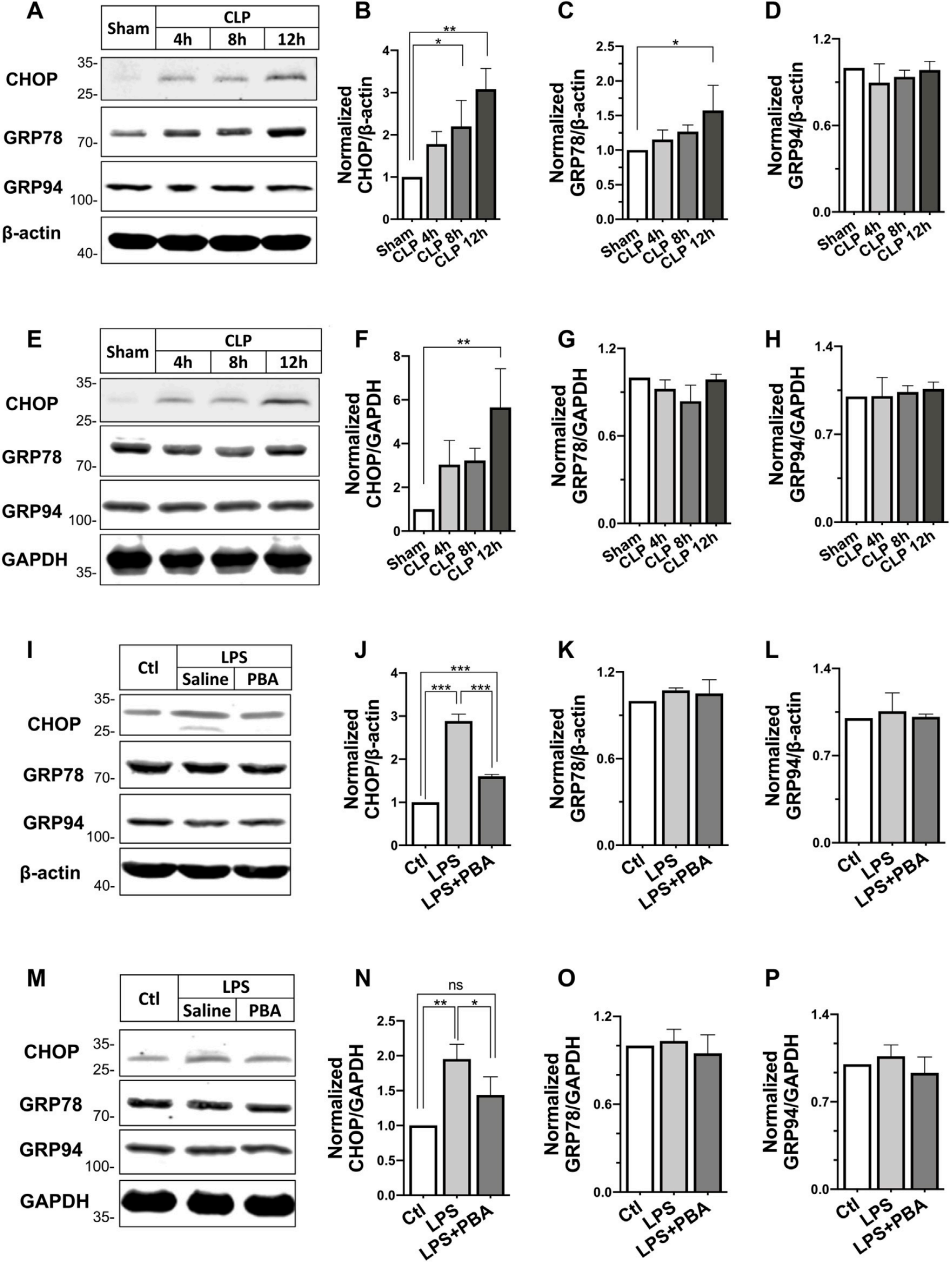
Effects of PBA on ERS-related protein expression after septic shock in rats. **(A–D)**, Changes in expression of C/EBP homologous protein (CHOP), glucose-regulated protein 78 (GRP78), glucose-regulated protein 94 (GRP94) in the superior mesenteric artery after different times of CLP (*n* = 3); **(E–H)**, Changes in expression of C/EBP homologous protein (CHOP), glucose-regulated protein 78 (GRP78), glucose-regulated protein 94 (GRP94) in the heart after different times of CLP (*n* = 3); **(I–L)**, effects of anti-ERS with PBA on the expression of CHOP, GRP78, and GRP94 in LPS-challenged VSMCs; **(M–P)**, effects of anti-ERS with PBA on the expression of CHOP, GRP78 and GRP94 in LPS-challenged H9C2 cardiomyocytes (*n* = 3). CLP, cecal ligation and puncture; CsA, cyclosporine A; PBA, 4-phenylbutyrate acid; **p < 0.01, ***p < 0.001; *ns*, not significant.

Besides, the expression of ERS-related proteins including C/EBP homologous protein (CHOP), glucose-regulated protein 78 (GRP78), and GRP94 in CLP-induced septic shock rats and LPS treated VSMCs and H9C2 cardiomyocytes were also investigated. Results showed the expression of CHOP was significantly increased in heart and SMA tissues after CLP in rats in a time-dependent manner, whereas the expression of GRP78 and GRP94 did not change significantly (Figures 5A–H). In VSMCs and cardiomyocytes, LPS incubation at 500 ng/ml increased CHOP expression significantly. Inhibition of ERS with PBA (5 μmol/L) abolished the LPS-induced increase in CHOP expression. LPS and PBA did not alter the expression of GRP78 and GRP94 (Figure 5I-P). These results suggested the ERS and opening of mPTP in H9C2 cardiomyocytes and VSMCs challenged with LPS.

### Contribution of Phenylbutyric Acid in Cyclosporine A-Induced the Inhibition of Mitochondrial Permeability Transition Pore Opening After Septic Shock

**FIGURE 6:**
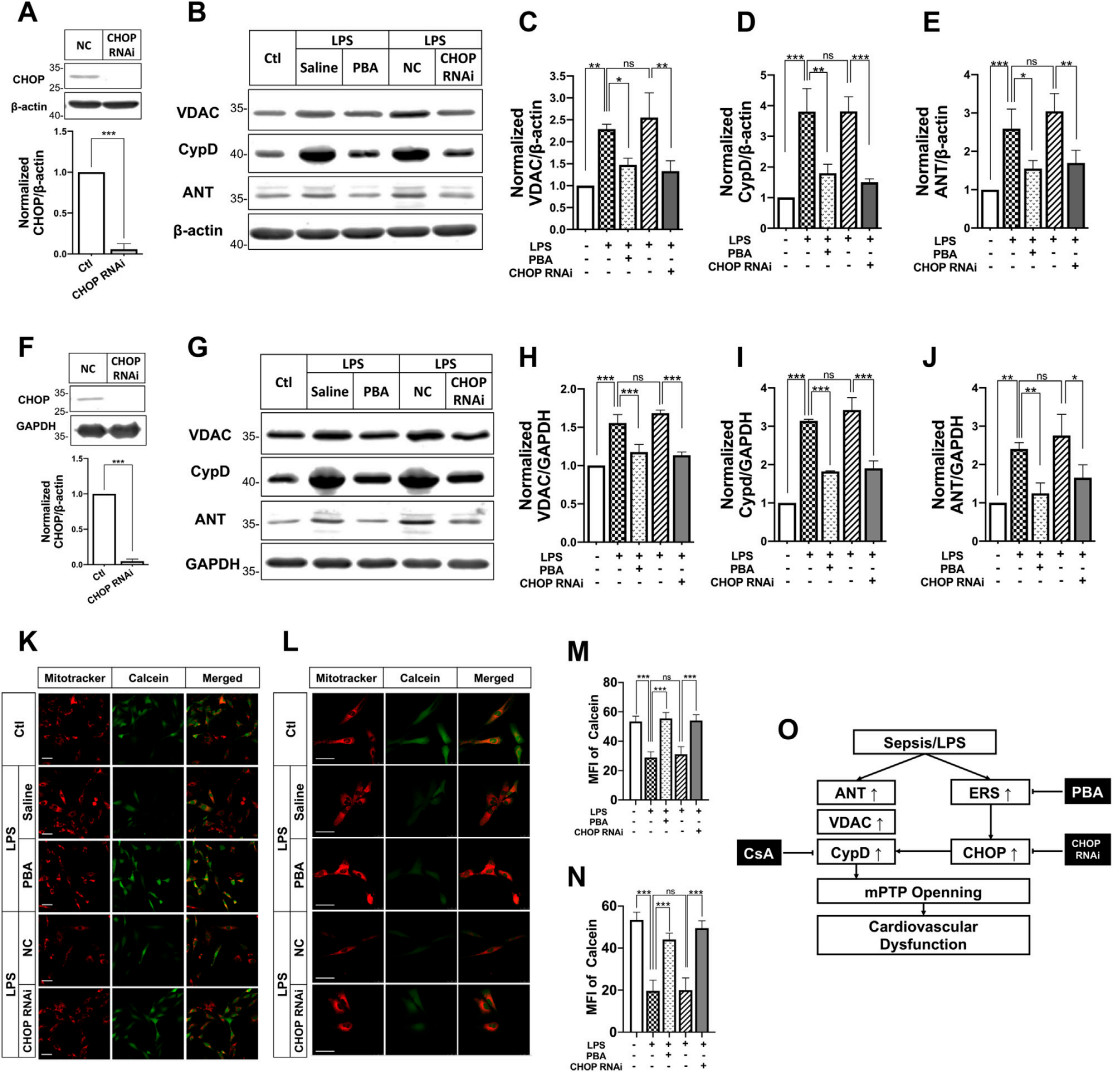
Effects of ERS on mPTP opening and its related protein expression after septic shock in rats in LPS-treated VSMCs and H9C2 cardiomyocytes. **(A)**, effects of CHOP RNAi on CHOP expression in VSMCs; **(B-E)**, effects of anti-ERS with PBA and CHOP-RNAi on the expression of mPTP-related proteins ANT, VDACs and CypD in VSMCs (*n* = 3); f, effects of CHOP RNAi on CHOP expression in H9C2 cardiomyocytes; **(G-J)**, effects of anti-ERS with PBA and CHOP-RNAi on the expression of mPTP-related proteins ANT, VDACs and CypD H9C2 cardiomyocytes (*n* = 3); **(K,M)**, effects of anti-ERS with PBA and CHOP-RNAi on mPTP opening in LPS-challenged VSMCs (*n* = 21/group from 3 independent experiment); **(L,N)**, effects of anti-ERS with PBA and CHOP-RNAi on mPTP opening in LPS-challenged H9C2 cardiomyocytes (*n* = 21/group from 3 independent experiment); **(O)**, schematic of synergetic effect of PBA and CsA protecting vascular function and benefiting to sepsis. Scale bar = 50 μm. CsA, cyclosporine A; PBA, 4-phenylbutyrate acid; MFI, mean fluorescence intensity. *p < 0.05, **p < 0.01, ***p < 0.001; *ns*, not significant.

To understand whether PBA affects mPTP opening after septic shock, the effects of PBA on mPTP opening were investigated in LPS-treated VSMCs and H9C2 cardiomyocytes. The results showed that treatment with PBA (5 μmol/L, 4 h) antagonized LPS-induced increases in expression of VDAC1, ANT1, CypD in VSMCs, and H9C2 cardiomyocytes (Figures 6A–J). Treatment with the ERS inhibitor PBA significantly reduced mPTP opening, and the fluorescence intensity of calcein was significantly increased compared with LPS treatment alone (Figure 6K-N).

The above study found that expression of CHOP was significantly increased after sepsis and PBA administration decreased the expression of CHOP. To further explore the relationship of ERS and mPTP opening, the effect of inhibition of CHOP (by transfection of its siRNA) on mPTP opening in VSMCs and H9C2 cardiomyocytes was observed. The results showed that interference of CHOP with its siRNA significantly inhibited the expression of CypD and ANT in VSMCs and H9C2 cardiomyocytes. Meanwhile, CHOP RNAi antagonized LPS-induced mPTP opening, too. The results further proved the close relationship between ERS and mPTP opening.

## Discussion

This study showed that inhibition of the mPTP opening with CsA or anti-ERS with PBA could improve cardiovascular function, increase the blood flow of vital organs, and increase the delivery and utilization of oxygen, and improve the survival in septic rats. The combination of PBA with CsA significantly enhanced the beneficial effects of CsA or PBA on both heart and blood vessels following septic shock. PBA enforced CsA-induced cardiovascular protection contributed the synergetic effect on sepsis treatment. The results indicated that PBA + CsA may be a perspective treatment for protecting cardiovascular function in sepsis or septic shock.

Despite the many efforts made for the treatment of sepsis or septic shock, the morbidity and mortality of severe sepsis and septic shock remained high. New therapeutic targets for sepsis have been proposed in recent years: protecting the glycocalyx, antioxidant (Uchimido et al., 2019), stimulating immune function, as well as blocking of cytokine storm (Tanaka et al., 2016; Venet and Monneret, 2018). However, searching for novel therapies is still important for reducing the mortality of sepsis or septic shock. This study found that inhibiting mPTP opening with CsA could decrease the morbidity of septic shock and increase animal survival in CLP-induced septic shock rats. Combining anti-ERS with PBA enforced the beneficial effects of CsA on septic shock rats, which suggested that anti-mPTP opening in combination with anti-ERS might be more effective strategy for the treatment of severe sepsis or septic shock.

In this study, we found that CsA benefited sepsis via inhibiting mPTP opening. CsA could interact with the hydrophobic pocket of CypD and inhibit PPIase activity of CypD and reduce mPTP opening. Besides, CsA would interact with CypD already bound to the ATP synthase and thereby protect mitochondria (Amanakis et al., 2020). What other mechanisms participate in the protective role of CsA in sepsis needs further investigation. For example, there were studies that showed that CsA was beneficial to impaired myocardial function following sepsis by blocking mitochondrial cytochrome c release and regulating calcineurin to mediate mitochondrial respiration and reduce tissue nitration and protein carbonylation (Fauvel et al., 2002; Joshi et al., 2006). CsA forms a complex with cytosolic cyclophilin-A that inhibits the calcium-activated protein phosphatase calcineurin, and through this pathway, the immune-suppressive effects were promoted (Schreiber and Crabtree, 1992) and CsA was commonly used in the clinic for the organ transplants and immunoregulation. Thus, the therapeutic application of CsA as a desensitizer of the mPTP has been hindered for many years. In our pilot study, five CsA doses (0.05, 1, 5, 10, 20 mg/kg) were applied; 5 mg/kg had better effects to inhibit mPTP opening, but whether 5 mg/kg CsA demonstrated immunosuppression, and whether PBA can inhibit or alleviate CsA-induced immune-suppression, is not known. And for the important role of mPTP in the progress of many diseases, several mPTP inhibitors have been developed for more cardiovascular protection and less adverse effects, such as sanglifehrin A, 6-MeAla-CsA, 4-methyl-val-CsA, N-methyl-4-isoleucine-CsA (NIM811), and D-3-MeAla-4-EtVal-CsA (Debio-025) and so on (Clarke et al., 2002; Hausenloy et al., 2003). Whether these compounds demonstrate immunosuppression effects and benefit sepsis remains to be explored.

The mPTP was opened in VSMCs and H9C2 cardiomyocytes after septic shock or stimulation with LPS. Several factors may lead to the opening of mPTP. Ca^2+^ overload in mitochondria, increases in oxidative stress, as well as depletion of adenosine triphosphate could result in the opening of mPTP. The endoplasmic reticulum and mitochondria are closely connected physically and functionally, Miki et al. noted that ERS in diabetic hearts impairs phosphoglycogen synthase kinase-3β-mediated suppression of mPTP opening (Miki et al., 2009; Belaidi et al., 2013). In this study, ERS also contributed to mPTP opening after septic shock. We found ERS in cardiac muscle and vascular smooth muscle after septic shock, indicated by the increase of CHOP expression in VSMCs and H9C2 cardiomyocytes after CLP or LPS stimulation. Inhibition of ERS with PBA prevented mPTP opening. Downregulation of CHOP by its RNAi antagonized LPS-induced mPTP opening as well as the increase in the expression of ANT and CypD. These results suggested that septic shock-induced ERS contributed to mPTP opening, and CHOP is the important molecule participating in this process.

PBA is an orally bioavailable terminal aromatic substituted fatty acid that is used in the treatment of various metabolic diseases, including cancer, genetic metabolic syndromes, neuropathies, diabetes, hemoglobinopathies, and urea cycle disorders. The mechanisms are varied. In general, PBA plays the role of an HDAC inhibitor or as a chemical chaperone rescuing conformational abnormalities of protein, and thus may help to reduce unfolded protein reaction and the ERS (Kusaczuk et al., 2015; He and Moreau, 2019). Previous studies reported that ERS act upstream of mPTP opening and thus in this study we investigated the effects of inhibition of ERS on mPTP opening. However, CsA was also reported to induce ERS vice versa (Rao et al., 2017). The mechanism of the synergetic effect might be explained as follows: a, PBA reduced the side effect of CsA and enhanced mPTP inhibiting; b, PBA reduced CypD expression through inhibiting ERS and CHOP (Figure 6O).

There might be other mechanisms that PBA enhanced the function of CsA, and we think nitric oxide (NO) might play a role. In severe sepsis, the content of plasma nitric oxide is significantly increased, and excessive nitric oxide could lead to impaired mitochondrial respiration (Vico et al., 2019) and mPTP opening in the heart (Dynnik et al., 2020). PBA was able to activate eNOS, increase NO level, and activate the mitochondrial mtNOS-SS system to inhibit mPTP opening (Dynnik et al., 2020). Increased NO level also improved the protective effect of CsA in the ischemia/reperfusion heart (Gross et al., 2013). These studies implied that the combination of PBA and CsA might benefit sepsis through NO, rather than just inhibiting ER stress.

In this study, the 72 h survival rate in the conventional treatment group was indeed similar to that in the untreated group. According to the guidelines for sepsis treatment, we administrated fluid resuscitation combined with antibiotics and vasoactive drugs for severe sepsis rats. At 6 h after treatment, 7/16 of the CT group survived, while only 3/16 of the untreated group survived, indicating that the conventional treatment was effective in the initial stage of treatment but advanced organ function protection measures were needed to further prolong the survival time. Our study has some limitations. Firstly, the effect of PBA on immunosuppression and kidney toxicity by CsA was unexplored. Secondly, although we found that ERS took part in the regulation of mPTP opening after septic shock, and CHOP may be the main molecule in this process, how CHOP regulates mPTP opening needs further investigation.

## Conclusion

The combined application of PBA and CsA produced synergetic protection against severe sepsis via improving cardiovascular function. The mechanism is closely related to PBA enforcing the inhibitory effect of CsA on mitochondrial permeability transition pore and reducing the side effect of CsA.

## Data Availability

The original contributions presented in the study are included in the article/Supplementary Material, further inquiries can be directed to the corresponding author.

## References

[B1] Amanakis G. Murphy E. Cyclophilin D. (2020). Cyclophilin D: An Integrator of Mitochondrial Function. Front. Physiol. 11, 595. 10.3389/fphys.2020.00595 32625108 PMC7311779

[B2] Belaidi E. Decorps J. Augeul L. Durand A. Ovize M. (2013). Endoplasmic Reticulum Stress Contributes to Heart protection Induced by Cyclophilin D Inhibition. Basic Res. Cardiol. 108 (4), 363. 10.1007/s00395-013-0363-z 23744057

[B3] Cecconi M. Evans L. Levy M. Rhodes A. (2018). Sepsis and Septic Shock. Lancet 392 (10141), 75–87. 10.1016/S0140-6736(18)30696-2 29937192

[B4] Clarke S. J. McStay G. P. Halestrap A. P. (2002). Sanglifehrin A Acts as a Potent Inhibitor of the Mitochondrial Permeability Transition and Reperfusion Injury of the Heart by Binding to Cyclophilin-D at a Different Site from Cyclosporin A. J. Biol. Chem. 277 (38), 34793–34799. 10.1074/jbc.M202191200 12095984

[B5] Duan C. Kuang L. Xiang X. Zhang J. Zhu Y. Wu Y. (2020). Drp1 Regulates Mitochondrial Dysfunction and Dysregulated Metabolism in Ischemic Injury via Clec16a-, BAX-, and GSH- Pathways. Cell Death Dis 11 (4), 251. 10.1038/s41419-020-2461-9 32312970 PMC7170874

[B6] Dynnik V. V. Grishina E. V. Fedotcheva N. I. (2020). The Mitochondrial NO-Synthase/guanylate Cyclase/protein Kinase G Signaling System Underpins the Dual Effects of Nitric Oxide on Mitochondrial Respiration and Opening of the Permeability Transition Pore. FEBS J. 287 (8), 1525–1536. 10.1111/febs.15090 31602795

[B7] Fauvel H. Marchetti P. Obert G. Joulain O. Chopin C. Formstecher P. (2002). Protective Effects of Cyclosporin A from Endotoxin-Induced Myocardial Dysfunction and Apoptosis in Rats. Am. J. Respir. Crit. Care Med. 165 (4), 449–455. 10.1164/ajrccm.165.4.2105084 11850335

[B8] Gross G. J. Hsu A. Pfeiffer A. W. Nithipatikom K. (2013). Roles of Endothelial Nitric Oxide Synthase (eNOS) and Mitochondrial Permeability Transition Pore (MPTP) in Epoxyeicosatrienoic Acid (EET)-induced Cardioprotection against Infarction in Intact Rat Hearts. J. Mol. Cel Cardiol 59, 20–29. 10.1016/j.yjmcc.2013.02.003 PMC3647061 23419451

[B9] Hausenloy D. J. Duchen M. R. Yellon D. M. (2003). Inhibiting Mitochondrial Permeability Transition Pore Opening at Reperfusion Protects against Ischaemia-Reperfusion Injury. Cardiovasc. Res. 60 (3), 617–625. 10.1016/j.cardiores.2003.09.025 14659807

[B10] He B. Moreau R. (2019). Lipid-regulating Properties of Butyric Acid and 4-phenylbutyric Acid: Molecular Mechanisms and Therapeutic Applications. Pharmacol. Res. 144, 116–131. 10.1016/j.phrs.2019.04.002 30954630

[B11] Hong J. Kim K. Kim J. H. Park Y. (2017). The Role of Endoplasmic Reticulum Stress in Cardiovascular Disease and Exercise. Int. J. Vasc. Med. 2017, 2049217. 10.1155/2017/2049217 28875043 PMC5569752

[B12] Joshi M. S. Julian M. W. Huff J. E. Bauer J. A. Xia Y. Crouser E. D. (2006). Calcineurin Regulates Myocardial Function during Acute Endotoxemia. Am. J. Respir. Crit. Care Med. 173 (9), 999–1007. 10.1164/rccm.200411-1507OC 16424445 PMC2662919

[B13] Khan M. M. Yang W. L. Wang P. (2015). Endoplasmic Reticulum Stress in Sepsis. Shock 44 (4), 294–304. 10.1097/SHK.0000000000000425 26125088 PMC4575622

[B14] Kusaczuk M. Bartoszewicz M. Cechowska-Pasko M. (2015). Phenylbutyric Acid: Simple Structure - Multiple Effects. Curr. Pharm. Des. 21 (16), 2147–2166. 10.2174/1381612821666150105160059 25557635

[B15] Leung A. W. Halestrap A. P. (2008). Recent Progress in Elucidating the Molecular Mechanism of the Mitochondrial Permeability Transition Pore. Biochim. Biophys. Acta 1777 (7-8), 946–952. 10.1016/j.bbabio.2008.03.009 18407825

[B16] Li T. Fang Y. Yang G. Xu J. Zhu Y. Liu L. (2011). Effects of the Balance in Activity of RhoA and Rac1 on the Shock-Induced Biphasic Change of Vascular Reactivity in Rats. Ann. Surg. 253 (1), 185–193. 10.1097/SLA.0b013e3181f9b88b 21233615

[B17] Li T. Fang Y. Yang G. Zhu Y. Xu J. Liu L. (2010). The Mechanism by Which RhoA Regulates Vascular Reactivity after Hemorrhagic Shock in Rats. Am. J. Physiol. Heart Circ. Physiol. 299 (2), H292–H299. 10.1152/ajpheart.01031.2009 20472763

[B18] Li T. Fang Y. Zhu Y. Fan X. Liao Z. Chen F. (2011). A Small Dose of Arginine Vasopressin in Combination with Norepinephrine Is a Good Early Treatment for Uncontrolled Hemorrhagic Shock after Hemostasis. J. Surg. Res. 169 (1), 76–84. 10.1016/j.jss.2010.02.001 20471036

[B19] Li T. Zhu Y. Liu L. (2010). Role of Rho Kinase in the Dysfunction of Myocardium Contraction in Hemorrhagic Shock Rats. Chin. J. Pathophysiol. 26, 1059–1063. 10.3969/j.issn.1000-4718.2010.06.004

[B20] Li T. Zhu Y. Tian K. Xue M. Peng X. Lan D. (2013). Ideal Resuscitation Pressure for Uncontrolled Hemorrhagic Shock in Different Ages and Sexes of Rats. Crit. Care 17 (5), R194. 10.1186/cc12888 24020401 PMC4264615

[B21] Liu L. Wu H. Zang J. Yang G. Zhu Y. Wu Y. (2016). 4-Phenylbutyric Acid Reveals Good Beneficial Effects on Vital Organ Function via Anti-endoplasmic Reticulum Stress in Septic Rats. Crit. Care Med. 44 (8), e689–701. 10.1097/CCM.0000000000001662 26958745

[B22] Miki T. Miura T. Hotta H. Tanno M. Yano T. Sato T. (2009). Endoplasmic Reticulum Stress in Diabetic Hearts Abolishes Erythropoietin-Induced Myocardial protection by Impairment of Phospho-Glycogen Synthase Kinase-3beta-Mediated Suppression of Mitochondrial Permeability Transition. Diabetes 58 (12), 2863–2872. 10.2337/db09-0158 19755525 PMC2780889

[B23] Morciano G. Bonora M. Campo G. Aquila G. Rizzo P. Giorgi C. (2017). Mechanistic Role of mPTP in Ischemia-Reperfusion Injury. Adv. Exp. Med. Biol. 982, 169–189. 10.1007/978-3-319-55330-6_9 28551787

[B24] Pool R. Gomez H. Kellum J. A. (2018). Mechanisms of Organ Dysfunction in Sepsis. Crit. Care Clin. 34 (1), 63–80. 10.1016/j.ccc.2017.08.003 29149942 PMC6922007

[B25] Rao S. R. Sundararajan S. Subbarayan R. Murugan Girija D. (2017). Cyclosporine-A Induces Endoplasmic Reticulum Stress and Influences Pro-apoptotic Factors in Human Gingival Fibroblasts. Mol. Cel Biochem 429 (1-2), 179–185. 10.1007/s11010-017-2945-9 28324237

[B26] Schreiber S. L. Crabtree G. R. (1992). The Mechanism of Action of Cyclosporin A and FK506. Immunol. Today 13 (4), 136–142. 10.1016/0167-5699(92)90111-J 1374612

[B27] Supinski G. S. Schroder E. A. Callahan L. A. (2020). Mitochondria and Critical Illness. Chest 157 (2), 310–322. 10.1016/j.chest.2019.08.2182 31494084 PMC7005375

[B28] Tanaka T. Narazaki M. Kishimoto T. (2016). Immunotherapeutic Implications of IL-6 Blockade for Cytokine Storm. Immunotherapy 8 (8), 959–970. 10.2217/imt-2016-0020 27381687

[B29] Uchimido R. Schmidt E. P. Shapiro N. I. (2019). The Glycocalyx: a Novel Diagnostic and Therapeutic Target in Sepsis. Crit. Care 23 (1), 16. 10.1186/s13054-018-2292-6 30654825 PMC6337861

[B30] van Gurp M. Festjens N. van Loo G. Saelens X. Vandenabeele P. (2003). Mitochondrial Intermembrane Proteins in Cell Death. Biochem. Biophys. Res. Commun. 304 (3), 487–497. 10.1016/s0006-291x(03)00621-1 12729583

[B31] Venet F. Monneret G. (2018). Advances in the Understanding and Treatment of Sepsis-Induced Immunosuppression. Nat. Rev. Nephrol. 14 (2), 121–137. 10.1038/nrneph.2017.165 29225343

[B32] Vico T. A. Marchini T. Ginart S. Lorenzetti M. A. Adán Areán J. S. Calabró V. (2019). Mitochondrial Bioenergetics Links Inflammation and Cardiac Contractility in Endotoxemia. Basic Res. Cardiol. 114 (5), 38. 10.1007/s00395-019-0745-y 31428876

[B33] Zanotti-Cavazzoni S. L. Hollenberg S. M. (2009). Cardiac Dysfunction in Severe Sepsis and Septic Shock. Curr. Opin. Crit. Care 15 (5), 392–397. 10.1097/MCC.0b013e3283307a4e 19633546

[B34] Zhang Q. Fu H. Zhang H. Xu F. Zou Z. Liu M. (2013). Hydrogen Sulfide Preconditioning Protects Rat Liver against Ischemia/reperfusion Injury by Activating Akt-GSK-3β Signaling and Inhibiting Mitochondrial Permeability Transition. PloS one 8 (9), e74422. 10.1371/journal.pone.0074422 24058562 PMC3772845

[B35] Zhu Y. Liu L. Peng X. Ding X. Yang G. Li T. (2013). Role of Adenosine A2A Receptor in Organ-specific Vascular Reactivity Following Hemorrhagic Shock in Rats. J. Surg. Res. 184 (2), 951–958. 10.1016/j.jss.2013.03.039 23587453

