# Erratum: Synergetic Effect of 4-Phenylbutyric Acid in Combination With Cyclosporine A on Cardiovascular Function in Sepsis Rats via Inhibition of Endoplasmic Reticulum Stress and Mitochondrial Permeability Transition Pore Opening

**DOI:** 10.3389/fphar.2021.845186

**Published:** 2022-01-18

**Authors:** Frontiers Production Office

**Affiliations:** Frontiers Media SA, Lausanne, Switzerland

**Keywords:** sepsis, cardiovascular function, synergetic effects, endoplasmic reticulum stress, mitochondrial permeability transition pore opening

Due to a production error, one of the corresponding author’s email addresses was written incorrectly. The correct email address for Liangming Liu is liangmingliu@yahoo.com. In addition, Tao Li was not marked as a corresponding author in the author list.

The publisher apologizes for this mistake. The original version of this article has been updated.

